# Eliminating exogenous insulin therapy in patients with type 2 diabetes by duodenal ablation and GLP-1RA decreases risk scores for cardiovascular events

**DOI:** 10.1186/s12933-022-01628-z

**Published:** 2022-09-22

**Authors:** S. Meiring, C. B. E. Busch, A. C. G. van Baar, R. Hemke, F. Holleman, M. Nieuwdorp, J. J. G. H. M. Bergman

**Affiliations:** 1 Gastroenterology and Hepatology, Amsterdam University Medical Centres, location AMC, Meibergdreef 9, 1105AZ Amsterdam, The Netherlands; 2 Department of Radiology and Nuclear Medicine, Amsterdam University Medical Centres, location AMC, Amsterdam, The Netherlands; 3 Department of Internal Medicine, Amsterdam University Medical Centres, location AMC, Amsterdam, The Netherlands; 4 Department of Internal and Vascular Medicine, Amsterdam University Medical Centres, location AMC, Amsterdam, The Netherlands

**Keywords:** Diabetes type 2, Duodenal ablation, DMR, Endoscopy, GLP-1 and duodenum

## Abstract

**Introduction:**

Duodenal Mucosal Resurfacing (DMR) is an endoscopic ablation technique aimed at improving glycaemia and metabolic health in patients with type 2 diabetes mellitus (T2DM). DMR has an insulin sensitizing effect in patients with T2DM. Reducing hyperinsulinemia can improve cardiovascular health. In the INSPIRE trial, we combined a single DMR with a glucagon-like-peptide-1 receptor agonist (GLP-1RA) and demonstrated elimination of insulin treatment in 69% of patients at 6 months and 53% of patients at 18 months while improving glycaemic control and metabolic health. We hypothesized that this treatment approach is associated with improved cardiovascular health, by reducing hyperinsulinemia.

**Methods:**

Before and 6 months after starting the combination treatment to replace insulin, the following assessments were performed to evaluate cardiovascular health: magnetic resonance imaging (MRI) to measure abdominal visceral adipose tissue volume, ambulatory 24 h blood pressure (ABPM) analysis, postprandial insulin and triglycerides, fasting lipid panel and urine microalbumin. The Atherosclerotic Cardiovascular Disease (ASCVD) score was calculated to estimate 10-year risk of cardiovascular disease or stroke and the diabetes lifetime-perspective prediction (DIAL) score was calculated to estimate years free of cardiovascular disease.

**Results:**

Six months after replacing exogenous insulin by DMR and GLP-1RA, visceral adipose tissue decreased significantly by 24%. Postprandial triglyceride and insulin concentrations decreased significantly (*p* < 0.001), as did total cholesterol (from median 3.64 (IQR 3.34–4.89) to 3.48 (3.18–3.97) mmol/l, *p* = 0.008), LDL (from median 1.92 (IQR 1.49–2.30) to 1.79 (1.49–2.08 mmol/l, *p* = 0.044), and urine microalbumin (from median 7 (IQR 3–27) to 4 (3–8) mg/l, *p* = 0.018). All daytime blood pressure values decreased significantly. The ASCVD 10-year risk score decreased (from median 13.6 (IQR 5.7–26.0) to 11.5 (4.2–22.5) %, *p* = 0.030)) and the DIAL score increased (from median 82 (IQR 81–83) to 83 (81–84) years, (*p* = 0.039)).

**Discussion:**

The combination of DMR and GLP-1RA to replace insulin therapy in patients with T2DM is associated with a positive effect on multiple parameters of cardiovascular health. Taken together, they show a pattern of overall improvement in cardiovascular health, as evidenced by decreased risk scores for cardiovascular complications. However, it is not yet clear whether these improvements will translate into a true reduction in cardiovascular events.

**Supplementary Information:**

The online version contains supplementary material available at 10.1186/s12933-022-01628-z.

## Introduction

Diabetes mellitus is an important public health challenge. Currently about 1 in 11 adults worldwide has type 2 diabetes mellitus (T2DM) and its prevalence is still rising [1]. Treatment of hyperglycaemia is paramount and multiple glucose lowering treatment options are established. Atherosclerotic cardiovascular disease (ASCVD), defined as coronary heart disease, cerebrovascular disease, and peripheral arterial disease, is the leading cause of morbidity and mortality for individuals with T2DM [2]. Consequently, hospitalization due to heart failure is twofold higher in patients with T2DM compared to the general population [3, 4]. Numerous studies demonstrated that controlling individual cardiovascular risk factors including hypertension, hyperlipidemia, and abdominal adiposity can prevent or slow down the progression of ASCVD in people with T2DM [5].

Although there is a variety of effective oral glucose lowering drugs, eventually treatment with exogenous insulin is necessary in many patients with T2DM. Unfortunately, hyperinsulinemia can elicit weight gain and further deterioration of metabolic health [6] and it does not treat insulin resistance, the root cause of T2DM. Therefore, finding alternatives for insulin therapy while maintaining glycaemic control in patients with T2DM is necessary.

Evidence is accumulating that the duodenum is an important regulator of glucose homeostasis and therefore a new target for the treatment of T2DM [7, 8]. Roux-en-Y gastric bypass (RYGB) surgery, which includes the bypassing of the duodenum, is a well-established treatment for T2DM and greatly improves insulin resistance. Patients undergoing RYGB surgery also demonstrate significant decreases in weight, body fat content, and plasma lipids [9–11].

Duodenal Mucosal Resurfacing (DMR) is a minimally invasive endoscopic procedure that ablates the duodenal mucosa with subsequent regeneration [12]. Data from animal model and human studies suggest that this is followed by an insulin-sensitizing effect that is similar to the metabolic improvements seen after bariatric surgery, but to a lesser extent [12–14]. In the INSPIRE pilot study we found that DMR combined with GLP-1RA successfully replaced insulin therapy in 69% (11/16) of patients with type 2 diabetes at 6 months and in 53% of patients at 18 months [14]. Moreover, improvements in glycaemic (HOMA-IR, fasting plasma glucose (FPG)) and metabolic parameters (weight, body fat and liver fat content) were seen in these patients.

In this sub study we investigated whether replacing exogenous insulin by the combination of DMR and GLP-1RA in these 16 patients also resulted in changes in cardiovascular health parameters and the 10-year risk score for cardiovascular disease.

## Materials and methods

### Study design and intervention

The INSPIRE study was a single-center, single-arm, prospective, open-label clinical study that evaluated the effect of a single DMR procedure combined with GLP-1RA (liraglutide), in patients with T2DM, treated with insulin therapy. The study protocol was approved by the medical ethics committee of the Amsterdam University Medical Center. The study was conducted in accordance with ICH Good Clinical Practice Guidelines and the Declaration of Helsinki. The study is registered under EudraCT number 2017-00,349-30 at Clinicaltrialsregister.eu. The primary endpoints of this study have been reported elsewhere [14]. This report presents the results of a pre-defined sub-study of the INSPIRE study investigating the changes in parameters of cardiovascular health after replacing insulin by DMR with GLP-1RA.

### Clinical study summary

We included 16 patients with T2DM using basal insulin, aged 28–75 years, with a body mass index of 24–40 kg/m2, a hemoglobin A1c (HbA1c) ≤ 8.0% (64 mmol/mol), and an adequate β-cell reserve (defined as fasting C-peptide > 0.5 nmol/l) [14]. Baseline characteristics can be found in Additional file 1: Table S1. The endoscopic DMR procedure was performed under deep sedation with propofol by a single endoscopist (JB) with experience in endoscopic DMR procedures. The DMR procedure involved circumferential hydrothermal ablation of the duodenal mucosa using an over-the-guidewire catheter, as described previously [12, 13]. Exogenous insulin administration was discontinued immediately after the DMR procedure. Patients were instructed to adhere to a 2 week post-procedural diet (i.e. gradual transition from liquid to solid food to allow adequate regeneration of the duodenal mucosa). After finishing the 2 week post-procedural diet, patients began with self-administration of subcutaneous GLP-1RA, liraglutide (Victoza®, Novo Nordisk A/S). Standard mild nutritional counselling and lifestyle education were provided before DMR and during follow-up [14]. All 16 enrolled patients underwent a successful DMR procedure defined as ≥ 5 sequential ablations of 2 axial centimeters each.

### Cardiovascular assessments

At baseline and 6 months after DMR multiple measurements were conducted to assess cardiovascular health and the risk of cardiovascular events. These assessments are listed below.

#### Visceral and subcutaneous fat volume measurements

During the clinical study, MRI images (MRI; model clinical 3 Tesla scanner, Achieva, Philips) were made to measure liver fat content at baseline and 6 months after DMR. We decided to use these available MRI images to measure abdominal visceral adipose tissue (VAT) and subcutaneous adipose tissue (SAT). Body composition measures are usually estimated at the level of lumbar vertebra 3 (L3) or 4 (L4) [15]. As these levels were not consistently available on the MRI examinations of the upper abdomen, measurements at one transversal slide closest to mid L2 were used. VAT and SAT segmentation was performed using manual outlining (mDixon fat images) and semi-automated thresholding using Radiant DICOM viewer (Medixant, Poznan, Poland) by an experienced radiologist. This method is considered the reference standard for the quantitative assessment of intra-abdominal adipose tissue.

#### Ambulatory blood pressure monitoring

Systolic and diastolic blood pressure, and heart rate were measured during 24 h using an ambulatory blood pressure monitoring (ABPM) device (IEM, Mobil-O-Graph NG ABPM Monitor) at baseline and at 6 months follow-up. The ABPM cuff was placed on the non-dominant arm unless there was a 20/10 mmHg difference between arms, in which case the arm with the higher reading was used. Patients were instructed to maintain normal activity during ABPM and to hold the arm still and at heart level during recording. Recordings were programmed for every 30 min during the day (07.00 to 22.00) and every 60 min during the night (22.00 to 07.00). After 24 h, the monitor was detached and returned to the hospital.

#### Lipid panel

At baseline and 6 months, blood was drawn to measure fasting total cholesterol, high density lipoprotein (HDL), low density lipoprotein (LDL) and triglycerides. Blood for total cholesterol, HDL, and triglyceride quantification was collected in a heparin tube at room temperature. Samples were brought to the laboratory for processing and analysis. Quantification was done using enzymatic colorimetric test performed on a Cobas c502 machine.

#### Postprandial insulin and triglycerides

All patients underwent a mixed meal tolerance test (MMTT) to measure postprandial plasma insulin and triglyceride concentrations at baseline and 6 months. All patients were using exogenous insulin at baseline and none were using exogenous insulin at the 6 month follow-up MMTT. If insulin was restarted in patients with an HbA1c > 7.5%, it was restarted after the 6 months follow-up visit. Patients were asked to ingest a liquid meal (Fresubin 200 ml, 2.0 kcal/ml) within 10 min. During the MMTT, blood samples were drawn at 0 min (fasting) and at 15, 30, 45, 60, 90, 120, 180, and 240 min following the start of the meal. Triglyceride quantification was performed with serum heparin tubes at room temperature with use of the enzymatic colorimetric test on a Cobas c702 machine. Insulin quantification was performed on SST tubes with gel and clot activator with use of the ILMA method on an Atellica (Siemens) machine.

#### Urine microalbumin

All patients delivered a urine sample at baseline and 6 months. Microalbumin quantification was performed with urine monovette tubes at room temperature with use of the immunoturbidimetry on a Cobas c502 machine.

#### 10-years cardiovascular risk score

Ten year risk of cardiovascular events was estimated with use of an online calculator of the ASCVD risk score (ACC/AHA ASCVD Risk Calculator (cvriskcalculator.com). The calculator has been validated and is based on the algorithm published by Goff et al. 2013 in the ACC/AHA Cardiovascular Risk Assessment Guidelines [16]. Calculation of the 10-year risk estimate for ASCVD risk can best be described as a series of steps, in which multiple calculated risks variables, based on pooled data, result in a combined risk estimate. The risk variables include age, gender, race, plasma cholesterol levels, blood pressure values, diabetes and smoking status, and the use of blood pressure-lowering medications.

#### Estimated life-years free of cardiovascular disease

Life-years free of cardiovascular disease were estimated by using the diabetes lifetime-perspective prediction (DIAL) model, consisting of two complementary competing risk adjusted Cox proportional hazards functions using data from people with T2DM registered in the Swedish National Diabetes Registry (*n* = 389,366) [17]. The risk variables include age, gender, geographic region, smoking status, history of ASCVD, duration of diabetes, insulin use, systolic blood pressure, body mass index (BMI), plasma cholesterol levels, HbA1c level, glomerular filtration rate (eGFR) and albuminuria values, cholesterol-lowering drug and anticoagulant use.

## Statistical analysis

Data are expressed as medians (interquartile ranges). The Wilcoxon paired signed-rank test was used to detect differences between baseline and 6 months follow-up of all parameters derived from the MRI (surface area VAT and SAT), fasting lipid panel (total cholesterol, HDL, LDL and triglycerides), urine microalbumin, ASCVD risk and DIAL score. Mixed effect models were used to detect differences between the calculations of 24 h ABPM assessment (24 h, per daytime, per nighttime, systolic and diastolic blood pressure, and heart rate), postprandial triglycerides and insulin concentrations at baseline and 6 months. The intervention was set as fixed effect whereas the time points and patient number were set as random effects. For the mixed effect model analyses, 12 measurements (evenly distributed) were used to assess 24 h ABPM, 12 measurements to assess daytime (07:00–22:00) and 10 measurements to assess nighttime (22:00–07:00) to compare systolic and diastolic blood pressure and MAP before and 6 months after DMR. Data was analyzed using SPSS IBM SPSS Statistics 25 (IBM, Armonk, New York, United States). Graphs were created with Graphpad Prism 8 (GraphPad Software Inc., La Jolla, California, United States). Statistical test were done two-sided with *p*-values ≤ 0.05 considered as statistically significant.

## Results

### Patient characteristics

All 16 enrolled patients underwent a successful DMR procedure. Patients were on average 61 years old, their T2DM duration was 11 years, and used 31 units of insulin per day prior to DMR. All baseline characteristics can be found in Additional file 1: Table S1. As mentioned in the introduction, 69% of patients (11/16) at 6 months and 53% (8/15) of patients at 18 months remained off insulin therapy with improved glycaemic control and improved parameters of metabolic health. Details on these clinical outcomes have been published previously [14].

### Abdominal visceral and subcutaneous fat volume decreased significantly

Cross-sectional area measurements of VAT were performed in 14/16 patients and for SAT in 13/16 patients, since one patient suffered from claustrophobia and did not undergo a second MRI and MRI slides of two patients did not include the L2 level. We observed a significant relative reduction in VAT (24.2%) and SAT (20.4%) at 6 months compared to baseline (Table 1).

**Table 1:** Abdominal VAT and SAT at baseline and 6 months follow-up, measured using MRI

	Baseline	6 months post DMR	*p*-value
VAT (cm^2^)	248 (184–294)	188 (156–244)	**0.002**
SAT (cm^2^)	152 (136–190)	121 (93–158)	**0.002**

Data are expressed as median (Q1-Q3)

Paired Wilcoxon signed-rank tests were used to compare measurements between baseline and 6 months

*DMR* duodenal mucosal resurfacing, *VAT* visceral adipose tissue, *SAT* subcutaneous adipose tissue, *MRI* magnetic resonance imaging

*P*-values ≤ 0.05 are displayed in bold

### Decrease in daytime blood pressure

ABPM was performed in all 16 patients. During the study period, one patient started with amlodipine 5 mg 3 months after DMR and one patient stopped hydrochlorothiazide 3 months after DMR. All other patients had no changes in their blood pressure lowering medication. Daytime systolic, diastolic and MAP decreased. Heart rate increased. The other values did not show significant changes. (Table 2).

**Table 2:** Overview of variables of 24 h ambulatory blood pressure monitoring. Data are expressed as median (Q1-Q3)

	Baseline	6 months post DMR	*p*-value
24 h systole (mmHg)	127 (114–141)	126 (114–136)	0.194
24 h diastole (mmHg)	79 (69–86)	77 (69–85)	0.325
24 h MAP (mmHg)	101 (91–111)	99 (92–108)	0.195
Daytime systole (mmHg)	132 (119–148)	127 (115–137)	**0.001**
Daytime diastole (mmHg)	83 (73–89)	79 (72–86)	**0.037**
Daytime MAP (mmHg)	104 (95–115)	100 (93–109)	** < 0.001**
Nighttime systole (mmHg)	121 (110–133)	121 (112–132)	0.667
Nighttime diastole (mmHg)	75 (65–81)	73 (64–83)	0.812
NIghttime MAP (mmHg)	97 (86–106)	96 (86–105)	0.554
24 h heart rate (beats/min)	78 (67–84)	81 (73–89)	** < 0.001**

Mixed effect models were used to compare measurements between baseline and 6 months post DMR

*DMR* Duodenal Mucosal Resurfacing, *24 h* 24 h, *mmHg* millimeter of mercury, *MAP* mean arterial pressure, *min* minute

*P*-values ≤ 0.05 are displayed in bold

### Fasting lipid panel improved

Fasting total cholesterol, LDL, and triglyceride concentrations significantly decreased at 6 months post DMR compared to baseline, whereas HDL concentrations did not change (Table 3).

**Table 3:** Fasting total cholesterol, HDL, LDL and triglyceride plasma concentrations at baseline and 6 months follow-up

	Baseline	6 months post DMR	*p*-value
Total cholesterol (mmol/l)	3.64 (3.34–4.89)	3.48 (3.18–3.97)	**0.008**
HDL (mmol/l)	1.21 (0.95–1.32)	1.15 (1.05–1.47)	0.103
LDL (mmol/l)	1.92 (1.49–2.30)	1.79 (1.49–2.08)	**0.044**
Triglycerides (mmol/l)	1.79 (1.15–2.66)	1.09 (0.91–1.89)	**0.023**

Data are expressed as median (Q1-Q3). Paired Wilcoxon signed-rank tests were used to compare measurements between baseline and 6 months

*HDL* high-density lipoprotein, *LDL*0 low-density lipoprotein

*P*-values ≤ 0.05 are displayed in bold

### Postprandial triglycerides and insulin decreased

**Fig. 1:**
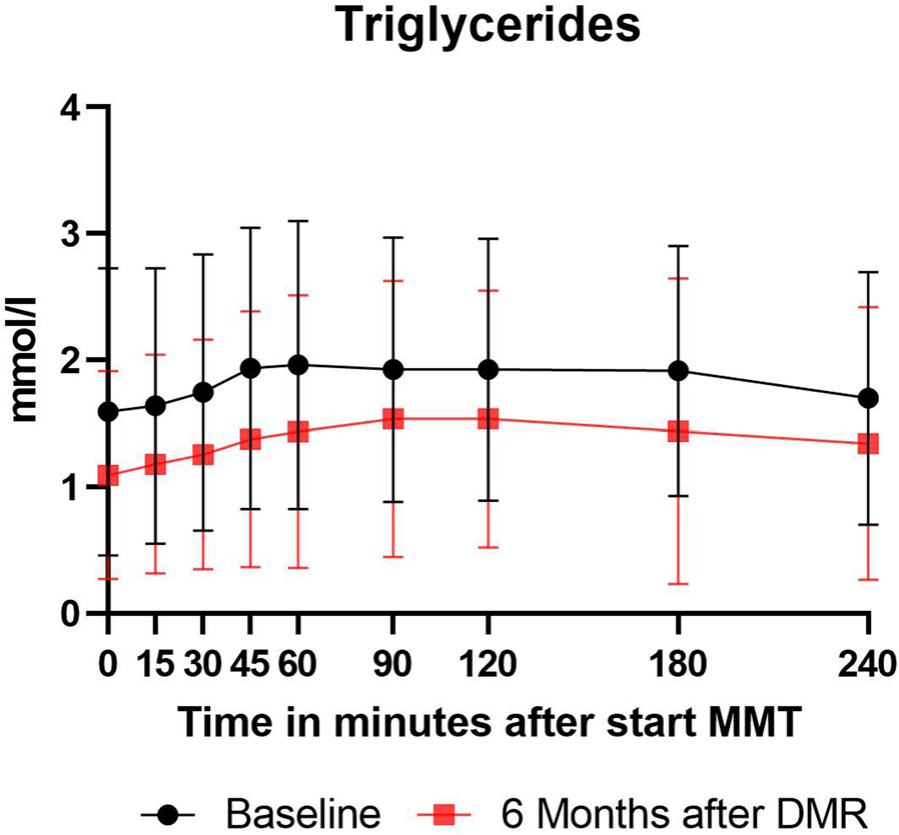
Postprandial triglyceride concentration curve during MMTT at baseline and 6 months in all 16 patients. Values are expressed as median (Q1-Q3). DMR: Duodenal Mucosal resurfacing, MMTT: mixed meal tolerance test

**Fig. 2:**
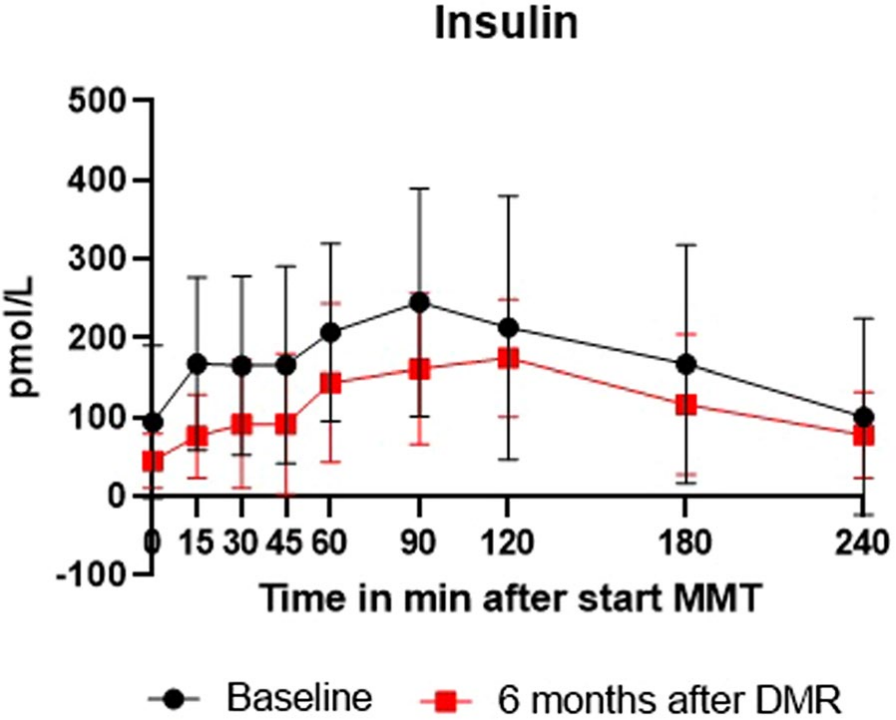
Postprandial insulin concentration curve during MMTT at baseline and 6 months in all 16 patients. Values are expressed as median (Q1-Q3). DMR: Duodenal Mucosal resurfacing, MMTT: mixed meal test

Postprandial triglyceride concentrations decreased significantly at 6 months post DMR compared to baseline, (*p* < 0.001, using mixed effect models) (Fig. 1). Postprandial insulin concentrations also dtecreased significantly, (*p* < 0.001)(Fig. 2).

### Urine microalbumin decreased

Urine microalbumin decreased significantly from 7 (3–27) mg/l at baseline to 4 (3–8) mg/l at 6 months post DMR, (*p* = 0.018).

### Cardiovascular risk score decreased and ASCVD-free years increased

The ASCVD risk score decreased significantly at 6 months after DMR. At baseline 6/16 (37.5%) patients had a ≥ 20.0% estimated 10-year risk of heart disease or stroke, this decreased to 4/16 (25%) at 6 months post DMR. (Table 4) The median ASCVD risk score also decreased significantly from 13.6 (5.7–26.0)% to 11.5 (4.2–22.5)%, (*p* = 0.030). In five patients the risk score lowered by a category. None of the patients increased in risk category. (Additional file 1: figure S1) The estimated ASCVD-free years, according to the DIAL model, increased significantly with one year, (*p* = 0.039). (Table 4).

**Table 4:** ASCVD risk score for estimated 10-year risk of heart disease or stroke

ASCVD Score	Baseline	6 months post DMR
	Number of patients (%)	Number of patients (%)
≥ 20.0% (High risk)	6 (37.5%)	4 (25%)
7.5–19.9% (Intermediate risk)	5 (31.25%)	7 (6.25%)
5.0–7.4% (Borderline risk)	3 (18.75%)	0
< 5.0% (Low risk)	2 (12.5%)	5 (18.75%)
Median risk score	13.6 (5.7–26.0)%	11.5 (4.2–22.5) %
Median ASCVD-free years (DIAL)	82 (81–83)	83 (81–84)

ASCVD risk score was assessed by the ASCVD algorithm using the following variables: gender, age, systolic blood pressure, total cholesterol and smoking status in our patient population at baseline and 6 months post DMR [16]. Data are expressed as number of patients (% of population). ASCVD Risk score Calculator can be found on ACC/AHA ASCVD Risk Calculator (cvriskcalculator.com) and DIAL score calculator on https://u-prevent.com/calculators/dialModel

*DMR* Duodenal Mucosal Resurfacing, *ASCVD* atherosclerotic cardiovascular disease, *DIAL* diabetes lifetime-perspective prediction

## Discussion

In this predefined sub-study we found that multiple cardiovascular parameters improved in patients with T2DM 6 months after replacing their insulin therapy with the combination of DMR and GLP-1RA. In line with this, the ASCVD risk score decreased significantly from 13.6% to 11.5% and in five patients the risk score even lowered by a category. This risk score provides an estimate of 10-year risk of cardiovascular events and is a recognized risk assessment for patients with T2DM [16]. In addition, by using the DIAL model, it was estimated that ASCVD-free life years increased by 1 year. The DIAL model was developed specifically for patients with T2DM [17]. Since cardiovascular complications are the leading cause of morbidity and mortality in individuals with T2DM, finding better ways to decrease this risk is desirable. In this article we demonstrated a positive impact of the combination of a single DMR and GLP-1RA on cardiovascular parameters.

The observed improvements in parameters of cardiovascular health in our patients are probably the result of two important changes that are part of our study intervention. Firstly, exogenous insulin therapy was discontinued. Hyperinsulinemia often leads to weight gain and further deterioration of metabolic health [6]. Secondly, the DMR procedure has been found to improve insulin sensitivity, also leading to lower levels of endogenous insulin [12–14, 18, 19]. The exact mechanism behind the insulin sensitizing effect of DMR has yet to be elucidated. We hypothesize that its insulin sensitizing effect can occur due to changes in either the gut-brain axis or local signalling to the liver and pancreas, cellular or histological changes in the duodenal mucosa, bile acid composition and microbiota diversity. In patients included in this INSPIRE study, we observed changes in bile acid composition [20] and minor changes in gut microbiota diversity [21]. We have taken duodenal biopsies before and 3 months after DMR to assess histological changes, this data is under evaluation.

We observed a significant relative reduction of abdominal VAT volume of 24%. Currently, there are no reference values available for VAT. However, excessive VAT is associated with cardiovascular morbidity [22, 23]. In a population at high risk, a lower VAT is therefore a logical positive outcome.

In addition, we assessed 24 h blood pressure. We found a significant decrease in daytime values at 6 months. Other values did not change, this could be due to the small sample size and the fact that night time values were already within normal ranges.

Plasma lipid optimization is an important part of cardiovascular risk management in patients with T2DM [24]. We observed that lipid levels improved in our study population 6 months after DMR. HDL remained stable, which is a positive finding as HDL levels are inversely correlated with cardiovascular disease [25].

Next, we observed that urine microalbumin decreased significantly. Urine microalbumin is an important gauge for renovascular health. We deem it unlikely that a major effect on nephrosclerosis was established in only 6 months. This decrease in microalbumin probably results from a decrease in hyperfiltration, which is common in patients with inadequately controlled T2DM.

This study has some limitations. Firstly, this is an observational uncontrolled proof-of-concept study with a limited sample size. Secondly, due to the design of the study, it is difficult to determine the effect of DMR or GLP-1RA separately. GLP-1RA therapy has been found non-inferior to glargine therapy in improving glycaemia, but solely in insulin-naïve patients [26]. Besides a significant short-term reduction of glycaemia, GLP-1RA therapy has been associated with a significant reduction of cardiovascular events in patients with T2DM, but only with a hazard ratio of 0.87 after 4 years [27]. Since the glycaemic and some of the metabolic effects of DMR and GLP-1RA appear to act synergistically, the combination may also lead to a greater and clinically more meaningful reduction of cardiovascular events. Larger randomized controlled trials are needed to confirm these findings and to evaluate the effect of DMR alone on parameters of cardiovascular health. Thirdly, VAT was measured at the level of L2, in contrast to what is advised in literature (L3-L4), however these slices were not available.

In conclusion, multiple parameters of cardiovascular health improved significantly in our study patients, 6 months after starting the combination of DMR and GLP-1RA to eliminate exogenous insulin therapy. Individual effects of these parameters might not be very impressive, but together they show a pattern of improvement in overall cardiovascular health, supported by absolute improvements in ASCVD and DIAL scores. In patients with T2DM with high risk of developing cardiovascular disease (based on the ASCVD algorithm), this combination treatment might be beneficial. It is not yet clear whether these improvements translate to a true reduction in cardiovascular events, but when controlled studies find similar results, this combination treatment could be a paradigm-shifting treatment approach in patients with T2DM at high risk of developing cardiovascular disease.

## Supplementary Information

**Additional file 1: Figure S1.** Individual ASCVD risk scores for estimated 10-year risk of heart disease or stroke per patient at baseline and 6 months after DMR. (16) Data are expressed as %. Calculator can be found on (ACC/AHA ASCVD Risk Calculator (cvriskcalculator.com). ASCVD: atherosclerotic cardiovascular disease, DMR: duodenal mucosal resurfacing. Patient baseline characteristics and medication use at study entry. Data is expressed as median (Q1-Q3). T2D: type 2 diabetes mellitus, BMI: body mass index, HOMA-IR: homeostatic model assessment for insulin resistance.

## Data Availability

The datasets used and/or analyzed during the current study are available from the corresponding author on reasonable request.

## Abbreviations

ABPM: Ambulatory 24 h blood pressure
ASCVD: Atherosclerotic cardiovascular Disease
BMI: Body mass index
DMR: Duodenal mucosal resurfacing
DIAL: Diabetes lifetime-perspective prediction
FPG: Fasting plasma glucose
GLP-1RA: Glucagon-like-peptide-1 receptor agonist
HbA1c: Hemoglobin A1c
HDL: High density lipoprotein
HOMA-IR: Homeostatic model of insulin resistance
ILMA: Immunoluminometric assay
LDL: Low density lipoprotein
L3: Lumbar vertebra 3
MAP: Mean arterial pressure
MMTT: Mixed meal tolerance test
MRI: Magnetic resonance imaging
RYGB: Roux-en-Y gastric bypass
SAT: Subcutaneous adipose tissue
SST: Serum-separating tube
T2DM: Type 2 diabetes mellitus
VAT: Visceral adipose tissue
