# Supplementary material for: Eliminating exogenous insulin therapy in patients with type 2 diabetes by duodenal ablation and GLP-1RA decreases risk scores for cardiovascular events
Source: Cardiovasc Diabetol. 2022 Sep 22;21:191. doi: 10.1186/s12933-022-01628-z (PMC9503196; doi:10.1186/s12933-022-01628-z)
Supplement: Supplementary file 1 — Additional file 1: Figure S1. Individual ASCVD risk scores for estimated 10-year risk of heart disease or stroke per patient at baseline and 6 months after DMR. (16) Data are expressed as %. Calculator can be found on (ACC/AHA ASCVD Risk Calculator (cvriskcalculator.com). ASCVD: atherosclerotic cardiovascular disease, DMR: duodenal mucosal resurfacing. Patient baseline characteristics and medication use at study entry. Data is expressed as median (Q1-Q3). T2D: type 2 diabetes mellitus, BMI: body mass index, HOMA-IR: homeostatic model assessment for insulin resistance. [file 12933_2022_1628_MOESM1_ESM.docx]

**Supplementary material**Supplementary table 1

| Patient characteristics (N=16) | |
| --- | --- |
| Age [years] | 61 (55–67) |
| Male gender, n (%) | 10 (63%) |
| Duration of T2D [years] | 11 (8–15) |
| Weight [kg] | 87.8 (80.2–99.7) |
| BMI [kg/m^2^] | 28.8 (26.5–31.7) |
| HbA1c [%], [mmol/mol] | 7.5 (7.1–7.9), 58 (54-63) |
| Fasting plasma glucose [mmol/l] | 10.1 (8.9–12.0) |
| Fasting plasma insulin [pmol/l] | 104 (49–178) |
| C-peptide [nmol/l] | 0.63 (0.55–0.91) |
| HOMA-IR | 8.4 (4.3–12.0) |
| Glucose-lowering medication | |
| Mean number of daily units of insulin | 31 (16–47) |
| Insulin, units per kg | 0.31 (0.20-0.50) |
| Insulin monotherapy, n (%) | 2 (12.5%) |
| Oral glucose lowering medications, n (%) | 14 (87.5%) |
| Metformin, n (%) | 13 (81.3%) |
| Empagliflozin, n (%) | 1 (6.25%) |

Supplementary figure 1

##### Individual ASCVD risk scores for estimated 10-year risk of heart disease or stroke per patient at baseline and 6 months after DMR. (16) Data are expressed as %. Calculator can be found on [ACC/AHA ASCVD Risk Calculator (cvriskcalculator.com)](https://www.cvriskcalculator.com/). Abbreviations: ASCVD: atherosclerotic cardiovascular disease, DMR: duodenal mucosal resurfacing.
